# A Machine Learning-Based Predictive Model for Predicting Lymph Node Metastasis in Patients With Ewing’s Sarcoma

**DOI:** 10.3389/fmed.2022.832108

**Published:** 2022-04-06

**Authors:** Wenle Li, Qian Zhou, Wencai Liu, Chan Xu, Zhi-Ri Tang, Shengtao Dong, Haosheng Wang, Wanying Li, Kai Zhang, Rong Li, Wenshi Zhang, Zhaohui Hu, Su Shibin, Qiang Liu, Sirui Kuang, Chengliang Yin

**Affiliations:** ^1^Department of Orthopedics, Xianyang Central Hospital, Xianyang, China; ^2^Clinical Medical Research Center, Xianyang Central Hospital, Xianyang, China; ^3^Department of Respiratory and Critical Care Medicine, The First People’s Hospital of Chongqing Liang Jiang New Area, Chongqing, China; ^4^Department of Orthopaedic Surgery, The First Affiliated Hospital of Nanchang University, Nanchang, China; ^5^Department of Dermatology, Xianyang Central Hospital, Xianyang, China; ^6^School of Physics and Technology, Wuhan University, Wuhan, China; ^7^Department of Spine Surgery, Second Affiliated Hospital of Dalian Medical University, Dalian, China; ^8^Department of Orthopaedics, The Second Hospital of Jilin University, Changchun, China; ^9^The First Clinical Medical College, Shaanxi University of Traditional Chinese Medicine, Xianyang, China; ^10^Department of Spinal Surgery, Liuzhou People’s Hospital, Liuzhou, China; ^11^Department of Business Management, Xiamen Bank, Xiamen, China; ^12^Faculty of Medicine, Macau University of Science and Technology, Macau, China

**Keywords:** Ewing sarcoma, lymph node metastasis, SEER, multi-center, machine learning, web calculator

## Abstract

**Objective:**

In order to provide reference for clinicians and bring convenience to clinical work, we seeked to develop and validate a risk prediction model for lymph node metastasis (LNM) of Ewing’s sarcoma (ES) based on machine learning (ML) algorithms.

**Methods:**

Clinicopathological data of 923 ES patients from the Surveillance, Epidemiology, and End Results (SEER) database and 51 ES patients from multi-center external validation set were retrospectively collected. We applied ML algorithms to establish a risk prediction model. Model performance was checked using 10-fold cross-validation in the training set and receiver operating characteristic (ROC) curve analysis in external validation set. After determining the best model, a web-based calculator was made to promote the clinical application.

**Results:**

LNM was confirmed or unable to evaluate in 13.86% (135 out of 974) ES patients. In multivariate logistic regression, race, T stage, M stage and lung metastases were independent predictors for LNM in ES. Six prediction models were established using random forest (RF), naive Bayes classifier (NBC), decision tree (DT), xgboost (XGB), gradient boosting machine (GBM), logistic regression (LR). In 10-fold cross-validation, the average area under curve (AUC) ranked from 0.705 to 0.764. In ROC curve analysis, AUC ranged from 0.612 to 0.727. The performance of the RF model ranked best. Accordingly, a web-based calculator was developed (https://share.streamlit.io/liuwencai2/es_lnm/main/es_lnm.py).

**Conclusion:**

With the help of clinicopathological data, clinicians can better identify LNM in ES patients. Risk prediction models established in this study performed well, especially the RF model.

## Introduction

Ewing’s sarcoma (ES) can be identified in bone and soft tissue (1). The main symptom of ES is local pain with or without paresthesia. Highly aggressive small round blue cell malignant neoplasm is a pathological feature of ES (2). 80% of ES patients are under the age of 20, with a male to female ratio of 1.5:1 (3). ES accounted for 3% of malignant tumors in children, which is the second most common malignant bone tumor in children (4).

ES has metastatic potentiality. It is a highly anaplastic, round cell tumor, primarily arising in the intramedullary portion of bone, poor prognosis and metastases are not uncommon. It is well established when imaging patients with primary bone malignancy that on occasions computed tomography (CT) scanning can offer unique information for the occurrence of metastasis. EWS-FLI1, a fusion protein identified in ES, played a key role in many transcription and translation processes, and may affect the initiation and progression of tumors (3). MMR pathway may be be associated with the proliferation, invasion and migration of ES tumor cells (2). There are some reports suggesting that the incidence of LNM in ES is higher than that in osteosarcoma and chondrosarcoma (5). Lungs are the most commonly involved part of the body (6).

Once ES has metastasized, the prognosis is poor. The location of ES is a very important factor in the prognosis. Even if the same tumor grows in different locations, the prognosis can vary greatly. In the past, multi-drug combination chemotherapy with surgery and radiotherapy increased its 5-year survival rate to 65–75% in a limited period of time. However, the 5-year survival rate in the metastatic period was usually less than 30% (3, 7, 8). The ability to accurately and non-invasively assess the risk of tumor metastasis has significant implications for treatment planning, post-operative follow-up and rehabilitation, precision medicine, and long-term public health policy (9–12).

LNM in ES is relatively uncommon. In addition to tumor stage, tumor size, primary location, age and treatment, regional lymph node involvement is a new independent adverse prognostic factor for ES (13). LNM can also contribute to risk stratification. The 5-year survival rate of ES decreased from 60.3 to 45.9% once LNM exist (5).

Predicting models of tumor LNM have been established in thyroid papillary carcinoma, bladder urothelial carcinoma and early esophageal squamous cell carcinoma (14–16). To our knowledge, there are no predicting models for LNM in ES. SEER database covers almost 30% population of the United States and is an major resource for the study of ES (17). Machine learning and medical big data have become a key step in the leap from evidence-based medicine to precision medicine, and there has been research, research, and research on a variety of diseases (18–21). More and more scholars are discovering the value of artificial intelligence and big data in medicine (12, 22, 23). Based on the data of ES in the SEER database, a model for predicting LNM was established by using the ML method and verified internally and externally.

## Materials and Methods

### Patients Population

By recruiting patients diagnosed with ES from 2010 to 2016 in the SEER database as a training set. Patients diagnosed with ES from 2010 to 2018 from the Second Affiliated Hospital of Jilin University, the Second Affiliated Hospital of Dalian Medical University, Liuzhou People’s Hospital, and Xianyang Central Hospital were included as the validation set. The inclusion criteria were listed as follows: (1) patients confirmed as primary ES with pathologically evidence; (2) ES patients with ICD-O-3/WHO 2008 morphology code 60; (3) clinicopathological information and survival time is complete. The exclusion criteria were listed as follows: (1) patients with other kinds of the primary tumor and unknown metastasis; (2) patients without available information such as clinicopathological and survival time. Given that the study was retrospective and the data came from an open database, informed consent was not used.

### Data Collection

Index such as race, age, sex, primary site, laterality, T stage, M stage, surgery, radiation, chemotherapy, bone metastases, lung metastases and survival time were collected both in the training set and validation set. In the training set, data were extracted using SEER * STAT (8.3.5) software. In the validation set, data were obtained and processed by two investigators independently. If there were any objections, the third investigator would participate in judgment. Microsoft spreadsheet (Microsoft spreadsheet, 2013, Redmond, United States) was utilized to check the consistency of all data.

### Statistical Methods

Continuous parameters following normal distribution were described by mean SD. Categorical parameters were presented as numerical values and proportions. Chi-square tests, Fisher’s exact tests, *t*-tests and logistic regression analysis were performed using R software (version 4.0.5). The difference was statistically significant when P < 0.05 with bilateral test. ML algorithms and web application were performed with the help of Python.

Demographic characteristics of the training set from SEER database and the validation set from multi-center were compared to identify the difference. We also divided the total study population into two sets based on the presence or absence of LNM and compared baseline information. To identify risk factors for LNM in patients with ES, we conducted univariate and multivariate logistic regression. Factors with *P* < 0.05 in the univariate logistic regression analysis were determined as variables for model establishment. RF, NBC, DT, XGB, GBM, and LR were performed in the training set to develop a prediction model. Analysis of relative importance ranking of each input variable was performed in each model. We adopted 10-fold cross-validation in the training set and ROC curve analysis in multi-center data to check the performance of each model. Define the model with the best performance based on the maximum AUC in internal and external validation. At last, a web-based calculator was appropriate to provide for clinical application of the final prediction model.

## Results

### Demographic Characteristics

A total of 974 ES patients were included in this study, of which 923 were from the SEER database and 51 were from the multi-center external validation set. Baseline data of the training set and the validation set were listed in Table 1. Race and radiation were two variables with *P* < 0.05. The main race was white race (81.8%) in the training set and other (yellow race, etc.) (100%) in the verification set. Radiation history accounted for 21.7% in the training set and 43.1% in the validation set. The differences were not statistically significant in all other indexes such as age, sex, primary site, laterality, T stage, M stage, surgery, chemotherapy, bone metastases, lung metastases and survival times.

**TABLE 1:** Baseline data table of the training and validation sets.

Variable	Level	Overall (*N* = 974)	Multi-center data (validation set, *N* = 51)	SEER (Training set, *N* = 923)	p
Race (%)	Black	38 (3.9)	0 (0.0)	38 (4.1)	< 0.001
	Other	126 (12.9)	51 (100.0)	75 (8.1)	
	White	810 (83.2)	0 (0.0)	810 (87.8)	
Age [median (IQR)]	NA	17.00 [12.00, 27.00]	17.00 [12.50, 30.50]	17.00 [12.00, 27.00]	0.453
Sex (%)	Female	415 (42.6)	23 (45.1)	392 (42.5)	0.823
	Male	559 (57.4)	28 (54.9)	531 (57.5)	
Lymph node metastases (%)	No	839 (86.1)	44 (86.3)	795 (86.1)	1
	Yes/Unable to evaluate	135 (13.9)	7 (13.7)	128 (13.9)	
Primary Site (%)	Axis bone	425 (43.6)	27 (52.9)	398 (43.1)	0.367
	Limb bone	317 (32.5)	13 (25.5)	304 (32.9)	
	other	232 (23.8)	11 (21.6)	221 (23.9)	
Laterality (%)	left	374 (38.4)	21 (41.2)	353 (38.2)	0.901
	Not a paired site	290 (29.8)	15 (29.4)	275 (29.8)	
	right	310 (31.8)	15 (29.4)	295 (32.0)	
T (%)	T1	345 (35.4)	20 (39.2)	325 (35.2)	0.008
	T2	429 (44.0)	25 (49.0)	404 (43.8)	
	T3	39 (4.0)	5 (9.8)	34 (3.7)	
	TX	161 (16.5)	1 (2.0)	160 (17.3)	
M (%)	M0	661 (67.9)	30 (58.8)	631 (68.4)	0.205
	M1	313 (32.1)	21 (41.2)	292 (31.6)	
Surgery (%)	No	407 (41.8)	25 (49.0)	382 (41.4)	0.352
	Yes	567 (58.2)	26 (51.0)	541 (58.6)	
Radiation (%)	No	752 (77.2)	29 (56.9)	723 (78.3)	0.001
	Yes	222 (22.8)	22 (43.1)	200 (21.7)	
Chemotherapy (%)	No/Unknown	57 (5.9)	0 (0.0)	57 (6.2)	0.128
	Yes	917 (94.1)	51 (100.0)	866 (93.8)	
Bone metastases (%)	No	826 (84.8)	40 (78.4)	786 (85.2)	0.27
	Yes	148 (15.2)	11 (21.6)	137 (14.8)	
Lung metastases (%)	No	791 (81.2)	41 (80.4)	750 (81.3)	1
	Yes	183 (18.8)	10 (19.6)	173 (18.7)	
Times [median (IQR)]	NA	26.00 [11.00, 47.00]	23.00 [12.50, 39.50]	26.00 [11.00, 47.00]	0.829

Baseline data of lymphatic metastases were shown in Table 2. In all study populations, there were a total of 135 cases with lymphatic metastasis or status that could not be assessed, including 128 cases from the training set and seven cases from the validation set. After comparing two sets, results revealed T stage, M stage, surgery, bone metastases, survival time was variables with P < 0.05.

**TABLE 2:** Patients baseline table of lymphatic metastases.

	Level	Overall (*N* = 974)	No (*N* = 839)	Yes/Unable to evaluate (*N* = 135)	*p*
Category (%)	Multicenter data	51 (5.2)	44 (5.2)	7 (5.2)	1
	SEER	923 (94.8)	795 (94.8)	128 (94.8)	
Race (%)	Black	38 (3.9)	27 (3.2)	11 (8.1)	0.022
	Other	126 (12.9)	108 (12.9)	18 (13.3)	
	White	810 (83.2)	704 (83.9)	106 (78.5)	
Age [mean (SD)]	NA	22.28 (16.34)	22.25 (16.38)	22.46 (16.17)	0.889
Sex (%)	Female	415 (42.6)	367 (43.7)	48 (35.6)	0.091
	Male	559 (57.4)	472 (56.3)	87 (64.4)	
Primary site (%)	Axis bone	425 (43.6)	363 (43.3)	62 (45.9)	0.62
	Limb bone	317 (32.5)	278 (33.1)	39 (28.9)	
	Other	232 (23.8)	198 (23.6)	34 (25.2)	
Laterality (%)	Left	374 (38.4)	325 (38.7)	49 (36.3)	0.621
	Not a paired site	290 (29.8)	245 (29.2)	45 (33.3)	
	Right	310 (31.8)	269 (32.1)	41 (30.4)	
T (%)	T1	345 (35.4)	320 (38.1)	25 (18.5)	<0.001
	T2	429 (44.0)	362 (43.1)	67 (49.6)	
	T3	39 (4.0)	34 (4.1)	5 (3.7)	
	TX	161 (16.5)	123 (14.7)	38 (28.1)	
M (%)	M0	661 (67.9)	605 (72.1)	56 (41.5)	<0.001
	M1	313 (32.1)	234 (27.9)	79 (58.5)	
Surgery (%)	No	407 (41.8)	335 (39.9)	72 (53.3)	0.005
	Yes	567 (58.2)	504 (60.1)	63 (46.7)	
Radiation (%)	No	752 (77.2)	645 (76.9)	107 (79.3)	0.616
	Yes	222 (22.8)	194 (23.1)	28 (20.7)	
Chemotherapy (%)	No/Unknown	57 (5.9)	47 (5.6)	10 (7.4)	0.527
	Yes	917 (94.1)	792 (94.4)	125 (92.6)	
Lung metastases (%)	No	791 (81.2)	712 (84.9)	79 (58.5)	<0.001
	Yes	183 (18.8)	127 (15.1)	56 (41.5)	
Bone metastases (%)	No	826 (84.8)	722 (86.1)	104 (77.0)	0.01
	Yes	148 (15.2)	117 (13.9)	31 (23.0)	
Times [mean (SD)]	NA	30.64 (22.64)	31.99 (22.73)	22.27 (20.23)	<0.001

### Univariate and Multivariate Logistic Regression Analyses

As shown in Table 3, logistics regression analysis was performed to define risk factors for LNM in ES. Firstly, clinicopathological characteristics of two sets were compared using univariate logistic regression analysis. Race, T stage, M stage, surgery, lung metastases were factors with *P* < 0.05 in univariate logistic regression analysis and entered multivariate regression analysis. Finally, we came to the conclusion that race (black, OR = 2.270, 95% CI = 1.020–5.052, *P* = 0.045), T stage (T2, OR = 1.733, 95% CI = 1.044–2.876, *P* = 0.033; Tx, OR = 2.712, 95% CI = 1.511–4.870, *P* = 0.001), M stage (M1, OR = 2.038, 95% CI = 1.157–3.591, *P* = 0.014), lung metastases (yes, OR = 1.877, 95%CI = 1.067–3.301, *P* = 0.029) were independent predictors for LNM in ES.

**TABLE 3:** Univariate and multivariate logistic regression analysis of risk factors for Lymph node metastases in patients with Ewing sarcoma.

Variables	Univariate OR (95% CI)	*p*-value	Multivariate OR (95% CI)	*p*-value
Age(years)	1.001 (0.990–1.012)	0.888	/	/
Race				
White	Ref	Ref	Ref	Ref
Black	2.706 (1.304–5.616)	0.008	2.270 (1.020–5.052)	0.045
Other	1.107 (0.646–1.898)	0.712	1.157 (0.655–2.043)	0.615
Sex				
Male	Ref	Ref	Ref	Ref
Female	0.710 (0.486–1.035)	0.075	/	/
Primary site				
Limb bones	Ref	Ref	Ref	Ref
Axis of a bone	1.217 (0.792–1.872)	0.370	/	/
other	1.224 (1.224–2.007)	0.423	/	/
Laterality				
Left	Ref	Ref	Ref	Ref
Right	1.011 (0.648–1.578)	0.962	/	/
Other	1.128 (0.787–1.887)	0.376	/	/
T				
T1	Ref	Ref	Ref	Ref
T2	2.369 (1.461–3.841)	0.000	1.733 (1.044–2.876)	0.033
T3	1.882 (0.677–5.237)	0.226	0.798 (0.270–2.362)	0.684
TX	3.954 (2.291–6.826)	0.000	2.712 (1.511–4.870)	0.001
M				
M0	Ref	Ref	Ref	Ref
M1	3.647 (2.509–5.302)	0.000	2.038 (1.157–3.591)	0.014
Surgery				
No	Ref	Ref	Ref	Ref
Yes	0.582 (0404–0.838)	0.004	1.127 (0.738–1.721)	0.581
Radiation				
No	Ref	Ref	Ref	Ref
Yes	0.742 (0.365–1.506)	0.408	/	/
Chemotherapy				
No	Ref	Ref	Ref	Ref
Yes	0.689 (0.348–1.366)	0.286	/	/
Lung metastases				
No	Ref	Ref	Ref	Ref
Yes	3.974 (2.688–5.875)	0.000	1.877 (1.067–3.301)	0.029

### The Performance of Machine Learning Algorithms

**FIGURE 1:**
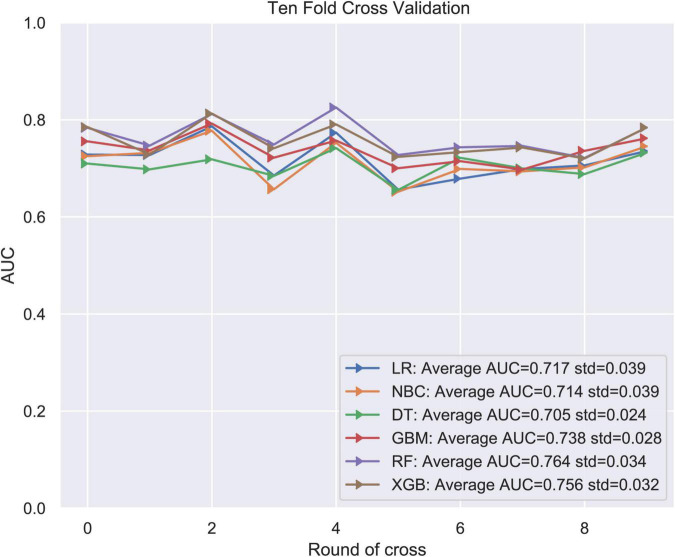
Ten-fold cross-validation of 6 ML algorithms for predicting LNM in patients with ES in the training set.

**FIGURE 2:**
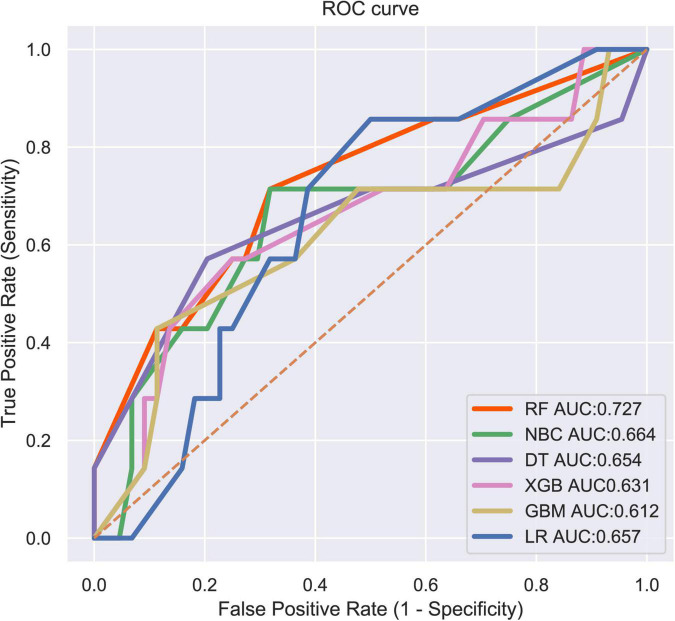
ROC curve analysis of 6 ML algorithms for predicting LNM in patients with ES in the validation set.

LNM status was regarded as the outcome index five factors with *P* < 0.05 in univariate logistic regression analysis mentioned above were defined as variables entered the model. ML algorithms including RF, NBC, DT, XGB, GBM, and LR were performed in the training set to develop prediction models. We adopted 10-fold cross-validation for internal validation to check the performance of each model (Figure 1). RF model performed best in predicting LNM in ES (average AUC = 0.764, std = 0.034). As shown in Figure 2, the RF model still showed the best performance in ROC curve analysis among 6 ML algorithms in the external validation (AUC = 0.727). Therefore, we chose the RF model as the final prediction model.

### Relative Importance of Variables in 6 Models

**FIGURE 3:**
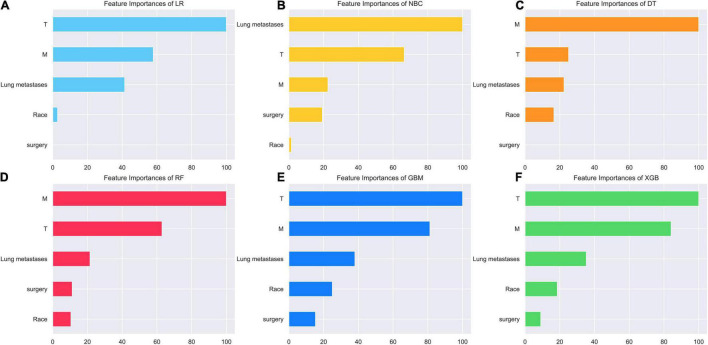
Relative importance ranking of each input variable for predicting models. **(A)** Random forest (RF). **(B)** Naive Bayes classifier (NBC). **(C)** Decision tree (DT). **(D)** Xgboost (XGB). **(E)** Gradient boosting machine (GBM). **(F)** Logistic regression (LR).

As we can see in Figure 3, the relative importance ranking of each input variable was slightly different among the 6 models. However, it was obvious that T Stage, M Stage, lung metastasis were the top three indicators in each model. Race and surgery were low-ranking variables. In the RF model, the relative importance rank of all variables from high to low was M Stage, T Stage, lung metastasis, surgery and race.

### Web-Based Calculator

**FIGURE 4:**
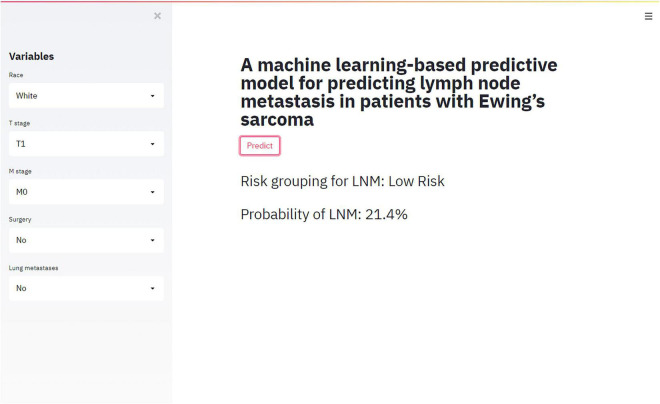
An example of the online calculator for predicting LNM in ES.

RF model performed best in 6 models. Accordingly, we established a web-based calculator to facilitate the clinical application of this prediction model (see text footnote 1; Figure 4).

## Discussion

ES is highly invasive, and LNM worsens the prognosis. This study included 923 cases of ES in the SEER database for model establishment and 51 cases of ES from four independent institutions in China for external validation. In the total study population, 135 cases of ES had LNM or unknown condition of lymph node involvement. Machine learning (ML) has emerged as a powerful computer-based method and as a “prediction tool” in medical domain. It has been applied to model clinical outcome to detect more interactions between variables and to improve cognition of tumor growth and progression (24–26). We adopted six ML methods: RF, NBC, DT, XGB, GBM and LR. RF model performed best. Importance from highest to lowest, M stage, T stage, lung metastases (LM), surgery and race were the five variables in the final model. Finally, we established an online network calculator to facilitate clinical application.

M stage was the most important variable in the model. The risk ratio for LNM in M1 patients was 2.038. At the initial diagnosis of ES, 15–30% of cases have multiple metastases. Even after treatment, 30–40% of ES patients still have local or distant metastases (27). Paulussen and colleagues studied 171 cases of primary metastatic ES and found the metastasis incidence of lung, bone, lymph node, brain and liver was 35.7, 37.4, 2.9, 1.2, and 0%, respectively (17). LNM is more common when ES occurs in extra-skeletal sties (5). Lymph node involvement may promote tumor progression. The invaded regional lymph nodes can play as metastasis stations for tumor cell proliferation (28).

T was factor next to M stage in importance ranking. Many studies have proposed a correlation between tumor size and LNM. Some scholars studied 73 patients of orbital sarcoma (eight cases of ES) and found a higher risk of LNM was related to disease category of at least T3 (OR = 13.33, 95%CI = 1.77–602.30, *P* = 0.004) (29). In previous studies of thyroid papillary carcinoma, bladder urothelial carcinoma, early esophageal squamous cell carcinoma, osteosarcoma, rhabdomyosarcoma, breast cancer and other tumors, the size of tumor is significantly related to lymph node involvement (30, 31). Edwards and others believe that lymphatic vessels are absent in normal bones or bone tumors but can be found in tumors that have extended to periosteum and surrounding soft tissue (28). Larger tumors may have invaded the periosteum and surrounding soft tissue, which may explain the relationship between tumor size and LNM.

Lung metastases was another critical indicator. ES frequently metastasizes to the lungs (6). In this study, the LNM risk ratio for ES patients with LM was 1.877. In our previous study, N stage was the most significant predictor of LM and about 30.8% of LM patients had N1 or Nx status. Similarly, the study of Kato Y suggested that the severity of lymph node involvement was strongly correlated with lung metastasis in colorectal cancer patients (32). The exact correlation mechanism between LM and LNM of ES patients is needed to be further revealed.

Surgery was also one of the indicators in the RF model. In univariate analysis, the surgery rate of patients whose LNM or status cannot be assessed was significantly lower than that of patients without LNM (46.7 vs. 60.1%, *P* = 0.005). The focus of ES may shrink after induction treatment. As a result, the micro focus may not be found by MRI. The residual living tumor cells at the primary tumor site may cause secondary metastasis. The current consensus is that all anatomical structures involved in the extension of the original pretreated tumor should be removed during surgery (33). In a Cox regression model of pelvic ES, complete resection of affected bone and disappearance of extraosseous tumor components were associated with a lower risk of death (33). Several studies have also shown that surgical resection of the primary tumor is significantly associated with improved overall survival (OS) in patients with metastatic primary bone ES (8).

Race remains an important predictor, even if the importance ranking is the lowest. The incidence rate of ES varies from race to race. The incidence rate of ES was higher in Asians and Caucasians than that in blacks (17). The incidence rate in China was 2–3 times lower than that in Europe and America (34). Compared with patients of other races, black ES patients had a lower 10-year survival rate and an increased incidence of metastatic diseases at diagnosis. ES larger than 10cm in Hispanic patients were more frequent (7). As we have previously mentioned, larger tumors are closely associated with LNM.

Since LNM of ES strongly affects prognosis, we strongly recommend the evaluation of suspicious regional lymph nodes. Fine needle aspiration cytology (FNAC) in the expanded regional scope with full evaluation is helpful for early diagnosis, resection and prognosis improvement (35). Sentinel lymph node biopsy (SLNB) can conduct targeted sampling of local lymph nodes to avoid radical surgery or random biopsy. However, the accuracy of SLNB will be reduced when performed in previously treated areas because distortion of lymphatic channels may lead to bypassing the real sentinel lymph nodes (31). FDG-PET scan can well identify LNM. However, the reliability of FDG-PET scan was lower than SLNB, especially for small volume metastatic lymph node diseases in sarcoma. This limitation was linked to possible false-positive uptake in the benign process (28, 36). Some scholars have developed an ES-specific probe named CS2-N-E9R with high sensitivity and selectivity for E/F fusion protein. There was a certain prospect of CS2-N-E9R for accurate identification of LNM (3). Patients with LNM can be regarded as candidates for new treatment strategies and clinical trials (28).

The innovation of this paper lies in the technical and methodological innovation. By using the machine learning method, we have performed better than other methods in terms of clinical data and its application. There were a few limitations in this study. Firstly, the LNM prevalence of ES in the SEER database may be underestimated (1). This suggests that more careful evaluation is important in order to improve the prognosis of ES patients. In addition, data on specific chemotherapy drugs and intensity, radiotherapy dose, detailed surgical information and treatment response in the SEER database is absent (17). Further, data from different modalities such as radiomics we did not include in the model, otherwise the model would have a more powerful predictive power (25, 37, 38). In the last, our validation cohort were all from China. Further multi-center, prospective and multi-ethnic validation is needed to test the effectiveness of the model.

## Conclusion

In this study, we applied ML algorithms to establish clinical risk models to predict LNM in ES patients. The RF model performed the best in internal and external validation. This model was an effective, non-invasive and convenient tool for clinical work. Further validation is needed.

## Data Availability Statement

The raw data supporting the conclusions of this article will be made available by the authors, without undue reservation.

## Ethics Statement

All participants signed written informed consents following the recommendations of the Institutional Review Board of the Xianyang Central Hospital. The ethical approval certificate is 20210022.

## Author Contributions

CLY, SRK, and QL designed the study. WLL collected and interpreted the data. WLL, QZ, and WCL drafted the manuscript. CX, ZRT, STD, HSW, WYL, and KZ provided expert consultations and clinical suggestions. RL, WSZ, ZHH, and SSB revised the manuscript. All authors reviewed the final version of the manuscript. All authors contributed to the article and approved the submitted version.

## Conflict of Interest

The authors declare that the research was conducted in the absence of any commercial or financial relationships that could be construed as a potential conflict of interest.

## Publisher’s Note

All claims expressed in this article are solely those of the authors and do not necessarily represent those of their affiliated organizations, or those of the publisher, the editors and the reviewers. Any product that may be evaluated in this article, or claim that may be made by its manufacturer, is not guaranteed or endorsed by the publisher.
